# Implementation of Quality by Design (QbD) Principles in Regulatory Dossiers of Medicinal Products in the European Union (EU) Between 2014 and 2019

**DOI:** 10.1007/s43441-020-00254-9

**Published:** 2021-01-13

**Authors:** Judith P. ter Horst, Sada L. Turimella, Frans Metsers, Alex Zwiers

**Affiliations:** Zwiers Regulatory Consultancy, Pivot Park, Kloosterstraat 9, 5349AB Oss, The Netherlands

**Keywords:** Quality by Design, QbD, EPAR, EMA, Drug development, Design space

## Abstract

**Background:**

Quality by Design (QbD) is a systematic risk-based approach to development, with predefined characteristics and quality risk management throughout the life cycle of a product. International Conference on Harmonization (ICH) guidelines Q8–Q11 give guidance on QbD applications with ICH Q8 (R2)—approved in 2009—describing the principles of QbD in detail. Since its adoption over 10 years ago, more information about QbD usage for the development of medicinal products is expected to be written in regulatory dossiers by companies.

**Methods:**

The present study set out to evaluate the implementation of QbD principles and elements in all EU approved marketing applications (MA) (*n* = 494), based on information available in the European Public Assessment Reports (EPARs), for a period of six years (2014–2019), starting 5 years after QbD adoption.

**Results:**

Of the 494 MAs, 271 were submitted with a full dossier (article 8(3)). According to EMA (38%), out of the 271 full dossier submissions, only 104 were developed using full QbD. This figure did not increase during this period. Interestingly, between 2014 and 2019, several MAs were not developed via full QbD implementation but used one or more QbD elements during development, including design space. In addition, a higher percentage of small molecule products were developed with QbD as opposed to biotechnology-derived products (78% vs. 22%, respectively).

**Conclusion:**

Overall, QbD during development of medicinal products is still not commonly described in dossiers. However, more companies started mentioning QbD elements, thus making it a promising step toward QbD as the standard for development in the future.

## Introduction

The aim of pharmaceutical development is to create a quality product and its manufacturing process should be designed to consistently deliver the intended performance of the product. Pharmaceutical companies use different strategies for product development: either by taking a conventional approach such as quality by testing, or systematically, such as Quality by Design (QbD) (see Table 1, [1]), or a combination of both.

**Table 1:** Product development: conventional approach versus QbD approach [1]

Aspects	Conventional	QbD
Pharmaceutical development	Empirical, typical single variable experiments	Systematic, multivariate experiments
Manufacturing process	Fixed	Flexible, changes can be made within design space
Process control	By in-process testing	Using process analytical technology (PAT) for feedback and feed forward in real time
Product specification	Based on previous experiences and batch data	Part of product performance in quality control strategy and checks
Control strategy	By either in-process quality or end product testing and inspection	Risk-based control strategy, real-time release

QbD is defined as a systematic risk-based approach for development that begins with predefined objectives. It focuses on product and process understanding and process control, and is based on sound science and quality risk management [2]. Application of QbD principles facilitate development of quality products and their assessment throughout their lifecycle, and ultimately result in greater patient benefit [3]. The basic principle of QbD is that quality cannot be tested into products, but that quality should be built in by design [2]. Therefore, QbD aims to ensure the desired quality of the product by assessing the variables which might impact the quality.

To ensure QbD, the ICH published the Q8 (Pharmaceutical Development) guideline in May 2006, which was supplemented between 2009 and 2012 by Q9 (Quality Risk Management [4]), Q10 (Pharmaceutical Quality System [5]) and Q11 (Development and Manufacture of Drug Substances [6]). In particular, the ICH Q8 (R2) guideline published in 2009 describes the principles of QbD which are Quality Target Product Profile (QTPP), Critical Quality Attributes (CQA), Quality Risk Management, Design Space, and Control Strategies (see Table 2) [2].

**Table 2:** Definitions of QbD elements [2, 4]

QbD principles	Definition
Control strategies	A planned set of controls, derived from current product and process understanding that ensures process performance and product quality
Critical process parameters (CPP)	A process parameter whose variability has an impact on a critical quality attribute and therefore should be monitored or controlled to ensure the process produces the desired quality
Critical quality attributes (CQA)	A physical, chemical, biological or microbiological property or characteristic that should be within an appropriate limit, range or distribution to ensure the desired product quality
Design space	The multidimensional combination and interaction of input variables (e.g., material attributes) and process parameters that have been demonstrated to provide assurance of quality
Proven acceptable range (PAR)	A characterized range of a process parameter for which operation within this range, while keeping other parameters constant, will result in producing a material meeting relevant quality criteria
Quality risk management	A systematic process for the assessment, control, communication and review of risks to the quality of the drug (medicinal) product across the product lifecycle
Quality target product profile (QTPP)	A prospective summary of the quality characteristics of a drug product that ideally will be achieved to ensure the desired quality, taking into account safety and efficacy of the drug product

Within the Pharmaceutical industry, it is generally acknowledged that the main benefits of QbD are increased process understanding, better control over product manufacturing and desired quality built into the development of the product (see Table 1). However companies who have to start with the implementation of QbD see higher initial costs and perceived regulatory hurdles as some of the drawbacks [7].

Since its adoption into the ICH guidelines Q8, Q9, Q10 and Q11 (over 10 years ago), pharmaceutical companies should have had sufficient time to implement QbD for the development of their products. However, in 2014 the European Medicine Agency (EMA) acknowledged that application dossiers with QbD information are far from becoming a standard approach, with only a relatively small number of marketing approval applications (MAA) made in Europe with supporting QbD data [8]. The present study aimed to evaluate the information about QbD usage in regulatory dossiers for the development of medicinal products approved in the EU for a period of 6 years, starting 5 years after the adoption date of ICH Q8 (2014–2019).

## Methods

The European Medicines Agency (EMA) website was evaluated for all products approved during the six-year period 2014–2019. The EMA publishes the European Public Assessment Report (EPAR) on its website, which provides public information of a medicine, including how it was assessed by EMA. For this publication, the EPARs of all approved products (published as the initial marketing document) in the above-mentioned period were evaluated for QbD principles incorporated into their development.

**Fig. 1:**
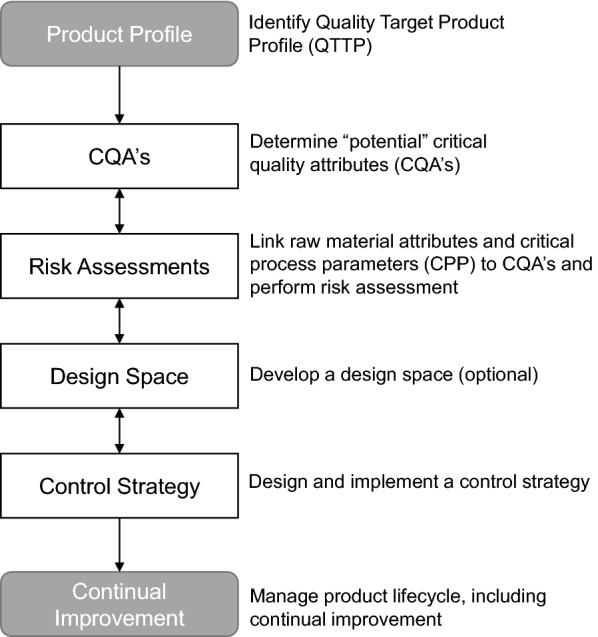
QbD scheme with QbD elements

Each EPAR was searched for the key word “Quality by Design” or QbD. All applications with this key word were classified as QbD applications unless explicitly specified by EMA that it was not a QbD application. Whether a medicinal product was categorized as “QbD” could depend on the judgment of the regulator and/or whether it had been specifically cited by the applicant. The QbD-containing applications were further searched for the QbD elements as mentioned in Fig. 1 and Table 2 [2, 4, 5].

The following type of data was also collected: the type of submission; special regulatory review processes or status; and type of products (small molecule/biotech-derived). The following biotech-derived products are included: antibodies, vaccines and cell therapies. siRNA products are classified as part of small molecule products because these are very complex chemical synthesized molecules.

Furthermore, data with regard to the size of company were also collected: small and medium-sized enterprise (SME) or large pharmaceutical companies. To qualify for a SME status, a company must employ less than 250 employees and have an annual turnover of not more than 50 million Euro. To confirm the SME status, the SME Register of the EMA was searched for the companies (https://fmapps.emea.europa.eu/SME/).

Per type of data collected, the percentage of QbD applications was calculated.

## Results

Firstly, the MAs of 494 medicinal products approved between 2014 and 2019 were ranked according to type of submission: full application (article 8(3)), well-established use (article 10a), fixed dose combinations (article 10b), informed consent (article 10c); and abridged applications with development versus a reference medicinal product: generic (article 10(1)), hybrid (article 10(3)), and similar biologic product (biosimilar, article 10(4)).

Table 3 shows the number of submission types for the medicinal products during 2014–2019 and the number and percentage of QbD used during their development. In total, 151 products of the 494 medicinal products were developed using QbD (31%). Out of the 494 approved medicinal products 271 were submitted with a full dossier (article 8(3)) and 104 of these were developed using QbD. Over this 6-year period, 30–50% of medicinal products approved via an article 8(3) submission were developed using QbD. Interestingly, approved fixed dose combination products were mostly developed via a QbD approach, starting at 50% in 2015 up to 100% in 2016 and 2019. As most of the medicinal products were approved via the legal basis of article 8(3) (*n* = 271) and not via article 10b (*n* = 24), it was chosen to continue with these products only.

**Table 3:** Type of EU submission and QbD development

	Total	Total QbD		2014	2014 QbD		2015	2015 QbD		2016	2016 QbD		2017	2017 QbD		2018	2018 QbD		2019	2019 QbD	
	*n*	*n*	%	*n*	*n*	%	*n*	*n*	%	*n*	*n*	%	*n*	*n*	%	*n*	*n*	%	*n*	*n*	%
All MAs	494	151	31	72	29	40	93	21	23	80	24	30	91	27	30	94	29	31	64	21	33
Art. 8(3)	271	104	38	42	22	52	56	18	32	44	16	36	38	12	32	54	20	37	37	16	43
Art. 10a	7	–	–	2	–	–	1	–	–	2	–	–	1	–	–	–	–	–	1	–	–
Art. 10b	24	14	58	8	2	25	4	2	50	2	2	100	5	4	80	3	2	67	2	2	100
Art. 10c	23	–	–	4	–	–	7	–	–	1	–	–	4	–	–	3	–	–	4	–	–
Art 10(1)	97	14	14	7	–	–	22	–	–	22	4	18	21	4	19	12	3	25	13	3	23
Art. 10(3)	28	10	36	6	5	83	3	1	33	5	2	40	6	1	17	6	1	17	2	–	–
Art. 10(4)	44	9	20	3	–	–	–	–	–	4	–	–	16	6	38	16	3	19	5	–	–

*Art.* Article, *MA* marketing authorisation

Art. 8(3): full application; art. 10a: well-established use; art. 10b: fixed dose combinations; art. 10c: informed consent; art. 10(1): generics; art. 10(3): hybrid; and art. 10(4): biosimilars

### QbD Elements

**Fig. 2:**
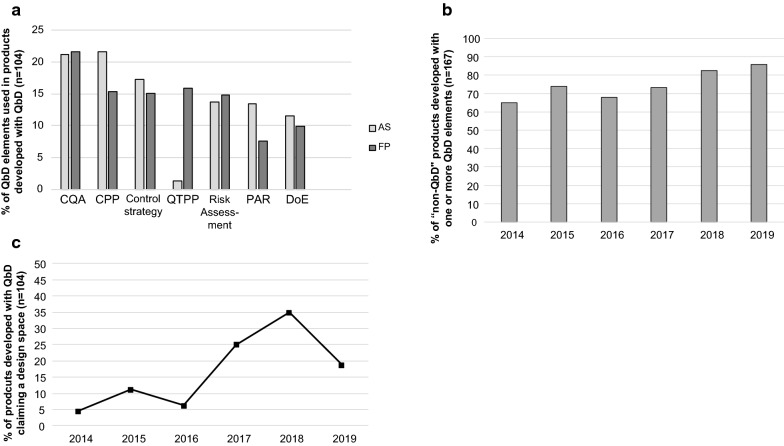
QbD elements. **a** Percentage of QbD elements used in medicinal products developed with full QbD for the period 2014–2019 (*n* = 104). Light gray bars represent QbD elements used during the development of the active substance (AS); dark gray bars represent QbD elements used during the development of the finished product (FP). **b** Percentage of products which are not developed with QbD according to EMA but using one or more QbD elements (*n* = 167). **c** Percentage of products developed with QbD claiming a design space. *AS* active substance, *CPP* critical process parameter, *CQA* critical quality attribute, *DoE* design of experiments, *FP* finished product, *PAR* proven acceptable range, *QTPP* quality target product profile

Next, the EPARs of article 8(3) submissions were searched for the following QbD elements: Quality Target Product Profile (QTPP), Critical Quality Attributes (CQA), risk assessment including failure mode effect analysis (FMEA), critical process parameters (CPP), design of experiments (DoE), proven acceptable range (PAR) and control strategy (Fig. 1, Table 2). Overall, there were only minor differences in QbD elements used between the active substance and finished product development phase in the medicinal products developed with QbD (Fig. 2a). The QTPP is the first step when starting with QbD and is, as expected, most commonly mentioned in the finished product section (16%) of the EPAR. Unexpectedly, in 1% it was observed in the active substance part without a cross-reference to the finished product part. It is not known whether this was due to an absence of the cross-reference, misuse of the term QTPP or misunderstanding of the concept. CPP and PAR are mentioned slightly more frequently in the active substance section of the EPAR than in the finished product part.

The next question was whether medicinal products which were not classified as fully developed with QbD, did use one or more of the QbD elements during the development. It was observed that, year on year, an increasing number of products were developed with the use of some QbD elements for both the active substance and the finished product, even though these products were not officially mentioned as using QbD in their EPARs (Fig. 2b). Claiming design space is another QbD element, but as this is optional it was not commonly implemented from 2014 to 2016 (Fig. 2c). However, between 2016 and 2018 an increase in claiming design space was seen from 6 to 35%.

### Small Molecule vs. Biotech Products

**Fig. 3:**
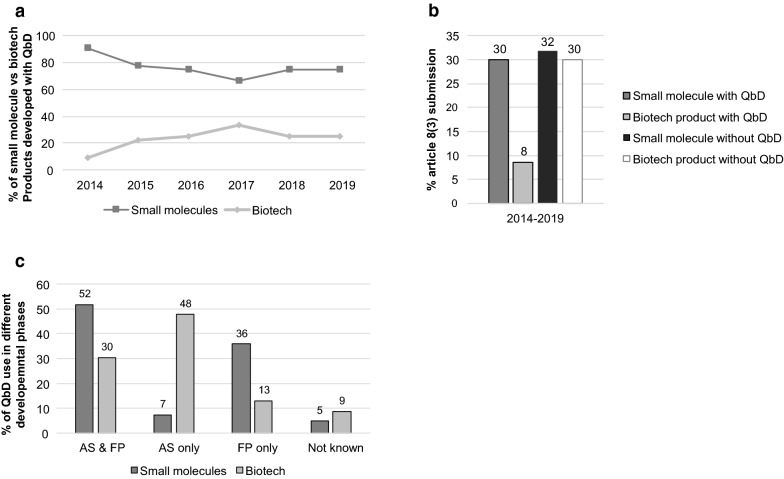
QbD in small molecule vs. biotech medicinal products. **a** Percentage of small molecule vs. biotech medicinal products developed with QbD for the period 2014–2019. **b** Percentage QbD usage of small molecules and biotech products submitted via full application. **c** Percentage of QbD during the different developmental phase of active substance and finished product for small molecules and biotech products. *AS* active substance, *FP* finished product

Most of the medicinal products submitted via a full application and developed with QbD are small molecule products as opposed to biotech products (78% vs. 22%, respectively, Fig. 3a). A small molecule product is developed by chemical synthesis, whereas biotech products are derived from living organisms such recombinant products, antibodies or vaccines. Three products were oligonucleotides created with RNA interference but since these products were chemically synthesized it was decided to define them as “small molecule”.

A small increase in biotech products using QbD for their development is seen from 2015 onwards (Fig. 3a). Overall, when analyzing all products submitted via full application during 2014–2019, it was observed that the same percentage of small molecule and biotech products do not use QbD for their development. This is comparable to the percentage of small molecule products which do use QbD (all around 30%, Fig. 3b), whereas only 8% of biotech products used QbD for their development.

The ICH guideline Q8 (R2) describes the QbD process specifically for drug product; ICH Q11 guides the QbD development of the active substance. It was conjectured whether QbD was used more during the development of active substance, the finished drug product, or both, and if there is a difference to be found between small molecule and biotech products. Figure 3c shows that 52% of small molecule products and 30% of the biotech products used QbD during the development of both the active substance and the finished product. 48% of the biotech products used QbD only during the active substance development compared to 7% of the small molecule products. QbD used only during the development of the finished product was seen in 36% of the small products and only in 13% of the biotech products.

### Special Regulatory Review Processes/Status

Could QbD development influence the chances of a medicinal product receiving a special review status over a product developed without QbD? To try to answer this, the EPARs of products submitted via a full application were examined for the following special regulatory review processes or statuses: orphan designation, accelerated assessment, conditional approval and exceptional circumstances. Most products did not qualify for a special review status. In those that did, it was observed that development with or without QbD had no effect.

### SME vs. Big Pharma

**Fig. 4:**
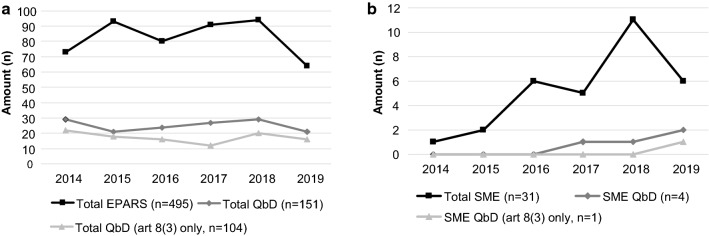
QbD usage in SMEs vs. large pharmaceutical companies. **a** Total EPARs analyzed over the period 2014–2019. Black line represents the total EPARs (*n* = 494), dark gray line represents total products developed with QbD (*n* = 151), light gray line shows the total products developed with QbD submitted via a full application (*n* = 104). **b** Total products submitted by a SME. Black line shows the total products (*n* = 31), dark gray line represents the total products developed with QbD by a SME (*n* = 4), light gray line shows the total products developed with QbD by a SME and submitted via a full application (*n* = 1). *SME* small and medium enterprises

SMEs developed only 31 out of the 494 medicinal products evaluated (Fig. 4). Out of the 151 products developed using QbD only four products were developed by SMEs starting from 2017 onwards. When focusing on the MAs submitted via a full application there was only one product submitted by a SME in 2019. The 104 products developed with QbD and submitted via a full application were made by 50 different companies (including one SME). Between the non-SME companies, there were no clear trends that certain companies applied QbD more frequently than others.

## Discussion

In 2014, EMA acknowledged that QbD applications are far from becoming a standard approach since a relatively small number of MAAs were made in Europe with supporting QbD data [8]. EMA reported that after averaging five submissions per year since 2008, QbD applications rose to eight submission in 2013 [8]. Here, the expectation of the authors was that in the coming years, more information about the usage of QbD would be found in the EPARs because there was more time for companies to implement QbD into their development. In particular, products that were in an early stage in the development process between 2009 and 2014 were in a unique position to participate fully in prospective QbD during development and were expected to go through the MAA review process from 2014 onwards. The present study has shown that, independent of submission type, only 151 out of 494 medicinal products approved in Europe during 2014–2019 described in their regulatory dossier the usage of QbD during the development of the product. Most of the approved medicinal products developed with QbD were submitted via a full application and with a complete dossier (*n* = 104). Compared to the eight QbD submissions made in 2013, from 2014 onwards, between 21 and 29 QbD applications were approved per year, which accounts for around 30% of all applications. Unfortunately, no increase in information about usage of full QbD in regulatory dossiers is seen from 2014 to 2019, especially for full applications. Fixed dose combination products are a positive exception however. In contrast to our results, Kajiwara et al. (2020) showed that in the PMDA reports in Japan the information about developmental ratio with QbD elements was increased from 9 to 71% between 2009 and 2018 [8]. Although full QbD development, as defined in the EPARs, is still not commonly described in most European regulatory dossiers, it is promising to see that more companies started to mention at least one or more QbD elements in their regulatory dossier as described in the guidelines ICH Q8-11, including claiming a design space. This latter element is an optional part of QbD and is not very often performed by the manufacturer since claiming a design space is challenging and somewhat questionable with respect to its return on investment [10]. In Japan, the development of design space for drug substances and products between 2009 and 2018 was even lower than in Europe with an average of 2% and no increase seen over time [9].

Although QbD is not mandatory, the expectation was that QbD would be applied more frequently by now since it has been over 10 years since the adoption of the ICH guidelines. Implementing QbD in development is expected to lead to an overall increase in quality of product which eventually will improve the trust and public image of the company [7]. In a QbD survey conducted in 2012, anonymous respondents from industry, academia and regulatory bodies reported high frequency (54% to 76%) of utilization of several tools and most QbD elements outlined by ICH Q8, with design of experiments, risk assessment, and the quality target product profile ranked as the top three [11]. So, what is it that holds companies back from describing the use of QbD elements during development in dossiers? Companies seem to be at very different places in terms of adoption of QbD. Some companies are skeptical about the idea of QbD, and therefore do not make much effort, whereas other companies have to start from scratch. It could be that the QbD “beginner” companies are not convinced of the business case. Also the regulatory benefits are unclear, due to the ICH guidance leaving room for flexibility and inconsistency of treatment of QbD by regulatory authorities [7]. Companies may also encounter many internal challenges as they attempt to implement QbD, such as internal misalignment and technical barriers when correct equipment is not available [12]. Some companies are more resistant to change. Furthermore, cultural issues, extra time and money, management issues and prioritization are substantial barriers for the execution of QbD [7]. Companies could believe that QbD is very costly and that it will slow down the development process, ultimately affecting the marketing authorization application—especially when there is competition for first filing of the product [7]. In addition, in 2012 more than 50% of respondents from industry were neutral about or disagreed with QbD leading to a better return on investment [11]. All of the challenges mentioned above, but most of all the costs needed to implement QbD for the first time, could explain why very few SMEs were found that implemented QbD into their development. Also, large pharmaceutical companies might have a sound infrastructure enabling them to incorporate QbD more easily.

This paper showed that more small molecule products than biotech products implemented QbD into their development. A typical biotechnology drug development process consists of a complex active substance development (which can include the development of master and working cell bank and manufacturing process, and scale-up); and a generally more straightforward finished drug product development, which can include the filling of the drug substance into the primary container [13]. Therefore, it is not surprising that when biotech products used QbD it was generally in the active substance development phase as opposed to the finished product phase. In 2015, a paper was published showing the insights and lessons learnt from discussions in the European Medicines Agency’s Biologicals Working Party and Committee for Medicinal Products for Human Use on the key issues during the evaluation of the marketing authorization of the first monoclonal antibody (a biotechnology-derived medicinal product) developed using extensive QbD concepts [14]. Before this evaluation, QbD was seen as an innovative approach to drug development that was started to be implemented into the regulatory framework mainly for chemical drugs. This paper demonstrated that implementing QbD for complex biotechnology products is feasible, but challenging, for both industry and regulators and that a common language and core understanding of principles and rules for consistency of approach and judgment is needed. Indeed, since 2014 a small increase in biotech companies using QbD was seen.

This research was performed by evaluating EPARs written and made available by the EMA. Without access to the full dossier the data of this paper is dependent upon what EMA wishes to publish but also what the company has decided to put into the regulatory dossier. Therefore, this assessment might not represent the actual implementation of QbD principles in the development of medicinal products but more how this information has been presented in dossiers in Europe. On a joint quality by design workshop of EMA and Parenteral Drug Association in 2014 regulators noted differences in terminology and the definitions used by pharmaceutical companies in their submissions, or during consultations on QbD matters [8]. This complicated the assessment and resulted in more questions by regulators, which could discourage companies to introduce QbD terminology into the regulatory dossiers even if QbD was applied in the development. This indicated the need for international harmonization of assessments, and specifically of terminology [8].

Another issue is that specific information on the development and usage of certain QbD elements (if used) of a known active substance could have been missed because the EPAR refers to the Certificate of Suitability (CEP) or the active substance master file (ASMF). For this reason, the data as presented in this paper should be seen as observational only.

## Conclusion

Based upon what is described in the EPARs, it seems that the use of full QbD during development of medicinal products is still not commonly described in regulatory dossiers. However, more companies are starting to experiment with the QbD concept and are developing mechanisms to support it—as seen by the adoption of one or more QbD elements in the development—making it a promising step toward QbD as the standard for development in the future. In spite of this, however, the pharmaceutical industry and the regulators still have a long way to go in order to make QbD a success story.
